# Stigma of infidelity associated with condom use explains low rates of condom uptake: qualitative data from Uganda and Tanzania

**DOI:** 10.1186/s12978-023-01563-6

**Published:** 2023-01-11

**Authors:** Kathryn Broderick, Christine Aristide, Brooke W. Bullington, Juliet Mwanga-Amumpaire, Jennifer A. Downs, Radhika Sundararajan

**Affiliations:** 1 Weill Cornell Medical College, 525 East 68th Street, Room M130, New York, NY 10065 USA; 2 Burlington Department of Epidemiology, Gillings School of Global Public Health, University of North Carolina at Chapel Hill, Chapel Hill, USA; 3 Carolina Population Center, University of North Carolina at Chapel Hill, Chapel Hill, USA; 4 Mbarara University of Science and Technology, Mbarara, Uganda; 5 Weill Cornell Medicine, Center for Global Health, New York, USA; 6 Weill Bugando School of Medicine, Mwanza, Tanzania

**Keywords:** Male condom, External condom, HIV/AIDS, Eastern Africa, HIV prevention

## Abstract

Despite widespread messaging supporting male (external) condom use to prevent HIV in endemic settings, utilization of condoms is low across sub-Saharan Africa. A thorough understanding of barriers to condom use as a form of HIV prevention is necessary to reduce HIV transmission. Here, we present qualitative data from rural eastern Africa to explain low utilization of condoms among heterosexual adults. Focus groups and interviews were conducted in Tanzania and Uganda between 2016 and 2019. A content analysis approach was used to identify attitudes about condoms and factors related to use/non-use. We found that strategies such as abstinence and being faithful to one’s partner are perceived as ideal but rarely achievable methods of HIV prevention. Condoms are used in the setting of “failure” to abstain or be faithful and are therefore stigmatized as markers of infidelity. As such, use within cohabiting and long-term relationships is low. Our data suggest that negative perceptions of condoms may stem from persistent effects of the formerly applied “ABC” HIV prevention approach, a public health messaging strategy that described A—abstinence, B—be faithful, and C—use a condom as tiered prevention tools. Condom uptake could increase if HIV prevention messaging acknowledges existing stigma and reframes condom use for proactive health prevention. These studies were approved by Weill Cornell Medicine (Protocols 1803019105 and 1604017171), Mbarara University of Science and Technology (Protocol 16/0117), Uganda National Council of Science and Technology (Protocol SS-4338), and the Tanzania National Institute for Medical Research (Protocol NIMR/HQ/R.8c/Vol.I/1330).

## Plain language summary

Condoms are used to prevent HIV infection. Even though public health organizations have encouraged people to use condoms, many people in sub-Saharan Africa do not, especially in sexual encounters with someone that they are living with or married to. In this study, we wanted to understand the reasons that people were not using condoms. Between 2016 and 2019, we spoke with individuals in Uganda in one-on-one interviews about HIV prevention and testing and with focus groups in Tanzania about family planning. We analyzed transcripts of these conversations to find common themes about people’s impressions of condom use. We learned that many of our participants believed that abstaining from sex and being faithful were the best ways to prevent HIV infection, but that they were not realistic strategies in the long term. Condoms were thought of as a useful tool for prevention when you “fail” at abstinence and monogamy. They were linked with being unfaithful, so people did not feel comfortable suggesting their use in committed relationships. These findings show that the “ABC” strategy for HIV prevention education may be continuing to make people think negatively about condom use. This strategy presented a tiered approach to HIV prevention, telling people it was best to (A) abstain, (B) be faithful to one’s partners, and (C) use a condom. In order to increase engagement with HIV prevention, public health messages need to acknowledge the negative associations between condoms and infidelity.

## Introduction

Sub-Saharan Africa accounts for 54% of the world’s HIV burden, though it is home to only 12% of the world’s population, according to the 2022 global UNAIDS update [1]. In this region, HIV is most frequently transmitted within heterosexual encounters, with a penile-vaginal intercourse transmission rate between 4 and 8 per 10,000 exposures [1–3]. While HIV incidence has declined across all populations over the past two decades, women and girls aged 15–49 continue to be disproportionately affected by new HIV infections in sub-Saharan Africa [1]. Poor engagement with prevention strategies, such as male (external) condom use, continues to be a barrier to epidemic control.

The majority of new HIV infections attributed to heterosexual sexual activity occur within cohabitating relationships, so effective HIV prevention strategies must have acceptability within long-term relationships [4]. Consistently used, male condoms decrease the risk of HIV transmission during penile-vaginal intercourse by at least 80%; however, condom uptake within cohabitating relationships is low in sub-Saharan Africa outside of known HIV serodiscordant couples [3, 5–7]. This low condom use has been attributed, in part, to women’s inability to negotiate condom use within marriage, particularly in traditionally patriarchal societies in which men have decision-making authority in households [8–11]. A 2011 qualitative study in Mozambique found that power differences between genders make it difficult for women to negotiate safer sexual practices with their male partners. Another 2011 study found that only 13.8% of married women in Malawi had ever used a condom with their marital partner [8, 12]. In contrast to cohabitating relationships, male condoms have been found to be more commonly used outside of marriages, with both men and women up to 18 times more likely to use male condoms in extramarital sexual encounters [13]. Condom use may, therefore, be low among cohabitating, long-term couples because they are associated with extramarital sexual encounters and mistrust of one’s partner [9, 13, 14].

The association of condoms with sexual mistrust may stem from the legacy of HIV prevention campaigns. In the 1990s, much of sub-Saharan Africa adopted the “ABC” public health model for HIV prevention: “Abstinence, Be faithful, and/or (use a) Condom.” Following the launch of ABC in Uganda, HIV incidence decreased from 15% in the early 1990’s to ~ 5% in 2001 [9, 15–17]. This dramatic reduction in HIV incidence was largely attributed to the ABC program, with abstinence and sexual partner reductions noted as the most impactful components of the strategy [9, 17]. These cornerstones have remained central to recent HIV prevention campaigns. For example, The United States’ President’s Emergency Plan for AIDS Relief (PEPFAR), enacted in 2006 and reauthorized in 2018, financially motivates abstinence-based prevention strategies by earmarking at least half of prevention funds for promoting abstinence, delay of sexual debut, monogamy, fidelity, and partner reduction [18, 19]. As with ABC, PEPFAR’s interventions include information on condom use, but emphasize reduction of sexual partners to prevent HIV transmission, stating “implementing partners must not give a conflicting message with regard to abstinence by confusing abstinence messages with condom marketing campaigns that appear to encourage sexual activity or appear to present abstinence and condom use as equally viable, alternative choices.” [19].

In the course of two different ongoing qualitative studies on HIV and family planning in Uganda and Tanzania, we observed that condoms were a stigmatized topic. The goal of this exploratory, qualitative analysis was to elucidate how perceptions and understandings of condom use may underpin low uptake of this prevention strategy in an HIV endemic region.

## Methods

### Study population

Qualitative data were collected in rural communities in eastern Africa. In Uganda, data were collected over the course of a multi-year study on engagement with HIV prevention in Mbarara District. In Tanzania, focus groups were conducted as part of a multi-year study on uptake of family planning in Mwanza. In Uganda, participants were recruited at outpatient healthcare practices. In Tanzania, participants were recruited in community settings including religious institutions, and health care providers were recruited at dispensaries.

### Data collection

In Uganda, 85 qualitative interviews were conducted between September 2017 and 2019. In Tanzania, 206 adult community members participated in 24 focus groups of ~ 8 individuals of the same gender, and 18 health care providers were interviewed one-on-one between October 2016 and September 2019. Interviews and focus groups were conducted in local languages (Runyankore in Uganda and Kiswahili in Tanzania) by Ugandan and Tanzanian research assistants who received training in qualitative data collection strategies. These discussions were audio recorded, transcribed, and translated into English. Research assistants conducting the data collection were of the same gender as the study participant(s). All participants were age ≥ 18 and provided written informed consent prior to participation. These studies were approved by Weill Cornell Medicine (Protocols 1803019105 and 1604017171), Mbarara University of Science and Technology (Protocol 16/0117), Uganda National Council of Science and Technology (Protocol SS-4338), and the Tanzania National Institute for Medical Research (Protocol NIMR/HQ/R.8c/Vol.I/1330).

### Data analysis

Qualitative interviews are used to explore lived experiences and individual perspectives on research topics, while focus groups intend to elicit group perspectives and consensus; therefore, these data collection strategies produce different types of qualitative data [20, 21]. In reviewing transcripts of these independent studies, authors RS and JD noted similarities in themes surrounding condom utilization. Overlapping content suggested that findings reflected both individual lived experiences as well as normative social experiences within our study populations. Following an inductive, content-analysis approach, all transcripts were re-reviewed to develop a coding scheme relevant to identify attitudes about condoms and factors that drive non-use [22]. Codes were independently developed by two authors (KB, RS) in vivo, through repeated engagement with the dataset. Coding schema were compared, with discrepancies in codes resolved through discussion until a consensus was reached. Using a framework approach [23], coded data were organized by topic and entered into an analytical matrix by author KB. Illustrative quotes were identified to represent dominant themes, and are presented here.

### Definitions of terminology

Sexual behavior terminology is fluid, with definitions influenced by factors such as respondent age and gender, setting of the interview, and cultural values [24]. In HIV prevention programs, abstinence has largely been conceptualized as delaying sexual activity until marriage, though “sexual activity” is not often explicitly defined. This lack of a precise definition for “sex” may contribute to ongoing HIV risk in the setting of perceived abstinence [3, 24]. Nonetheless, given the ubiquity of messaging using the terms “abstinence” and “sex” in the context of HIV prevention in the region, we use them in our analysis. While the terms “male condom” and “external condom” are both used in the literature, we use the term “male condom” here in alignment with both the convention of the field and the diction of our participants. These constructs are shaped by the sociocultural milieu of our participants, who reside in rural sub-Saharan Africa. Here, “sex” is defined as heterosexual, penetrative intercourse. “Abstinence” is conceived as absence of sex, and “stable relationships” as relationships in which monogamy, or “being faithful” is presented as the ideal. This includes marriages, cohabitating relationships, and other long-term committed partnerships.

## Results

### Characteristics of study participants

In Uganda, 54% of participants were female (*n* = 30). Median age was 36 years old. In Tanzania, 50% of participants were female (*n* = 104). Median age was 40 years old. Overall, our qualitative data point to the idealization of abstinence and monogamy, and illustrate how stigma associated with external condom use leads to low condom uptake in eastern Africa. We observed that participants describe abstinence and monogamy as optimal, but unrealistic, forms of HIV prevention; condoms are used in the setting of “failure” to achieve these ideals. As such, condoms are consistently perceived as intended for use during casual or extramarital relationships and therefore are highly stigmatized as markers of infidelity. We discuss these concepts in more detail below and provide illustrative quotes from our dataset as examples.

### Abstinence and monogamy are not realistic HIV prevention strategies

Participants reported having previously received counseling and education that abstinence was the ideal strategy to prevent HIV infection.

*“They asked me not to be promiscuous and to avoid sexual relationships.”—male, interview, Uganda.*

However, abstinence was largely acknowledged as unsustainable. In light of “failing” to be abstinent, condom use was described as a less optimal approach.

*“After receiving [HIV prevention] counseling… I managed to abstain for two years, but later failed and resorted to condom use [in casual relationships]”—male, interview, Uganda.*

Among those in long-term relationships, participants endorsed that “being faithful” to one’s partner was an optimal approach to mitigate HIV risk. However, many acknowledged that they remained at personal risk of HIV acquisition in these relationships. They did not consider stable relationships to be protective against HIV. In some cases, sexual partners were known to be non-monogamous.

*“I had a wife who was promiscuous, and I separated from her. I decided to go for an HIV test because I was concerned that she could have infected me with HIV.”—male, interview, Uganda*

The sexual behavior of one’s partners was described as a significant HIV risk factor outside of one’s own control. Participants admitted that they assumed the HIV transmission risk associated with their partner’s—often unknown—sexual activities. Women, in particular, cited difficulty calculating their level of risk due to their partners’ unknown behavior and an inability to reduce risk by negotiating terms of sexual behavior within long-term relationships.

*“I do not know [my husband’s] sexual-related behaviors. I cannot tell whether he sleeps with infected women or not. I cannot tell who he spends time with. I myself I do not have other sexual partners, but I am not sure of my husband. With those reasons, I am at high risk of contracting HIV”—female, interview, Uganda*

*“I also asked them how I can protect myself from HIV because we are four people in our marriage, If I protect myself from HIV when the others are not protecting themselves from HIV, how will it help?”—female, interview, Uganda.*

These data illustrate perceptions of abstinence and monogamy as unrealistic strategies. Even in relationships intended to be monogamous, the behaviors of a sexual partner are outside of an individual’s own control and therefore a source of significant risk for HIV acquisition.

### Male condoms are associated with infidelity

Though participants were cognizant of a real HIV risk within their sexual relationships, male condoms were not typically utilized within stable relationships as HIV prevention or family planning. Instead, condoms were overwhelmingly reported to be used in casual or extramarital relationships.

*“I do not sleep with different men without using condoms. I only sleep with my boyfriend without a condom”—female, interview, Uganda*

*“A few [people] use [condoms] as part of family planning, but most of them use them outside their marriages”—male physician, interview, Tanzania*

Condoms were described as useful for preventing HIV in the context of these sexual relationships when a sexual partner’s HIV status was unknown.

*“You can use condoms during sex when you are going to have sex with a woman whose HIV status is unknown to you.”—male, interview, Uganda*

Condom use was also specifically viewed as an important part of HIV prevention in the context of extramarital affairs, though primarily among men where extramarital intercourse tended to be normalized.

*“This is good advice because you can cheat on your wife when using condoms and you remain protected from contracting HIV”—male, interview, Uganda*

Participants described receiving HIV education that specifically emphasized use of condoms when engaging in sexual activity outside of stable relationships. The same HIV prevention strategy did not apply to intercourse with one’s spouse.

*“I should use a rubber for sex on the side to prevent disease, but I haven’t been told to use it during sex with my wife”—male, focus group, Tanzania*

*“They told me that I should be sexually faithful to my wife but, if I can’t be faithful to her, I can use a condom”—male, interview, Uganda*

In fact, condoms were so strongly associated with infidelity that possessing a condom stigmatized the person who possessed them as unfaithful.

*“Because I am respected, I cannot meet someone I know and still take condoms. They will perceive me badly because I have a wife.”—Female nurse, interview, Tanzania, describing her male clients’ reluctance to take condoms that were distributed freely at the local dispensary*

These data exemplify how condoms are conceptualized as HIV prevention pertinent to specific sexual encounters: namely, that their use is appropriate when the partner’s HIV status is unknown and/or with casual sexual contacts. In no instance did we find condoms described as a component of intercourse within stable relationships.

## Discussion

Our data illustrate that low condom use in eastern Africa can be attributed to the mixed messaging of HIV prevention campaigns, specifically ABC, which stigmatize male condoms by linking them with infidelity and mistrust. We have shown that distrust of one’s partner is a primary driver for condom use, leading people to use condoms with casual sexual partners, but not within partnerships where sexual exclusivity—or ‘being faithful’—is idealized.

### Stigmatization of condoms in stable relationships

Condoms were not widely endorsed as a tool for family planning and were not a “normal” part of stable relationships. Women in our study cited an inability to protect themselves from the risk of their male partners’ extramarital sexual encounters—which were normalized as part of long-term relationships. Condom use relies on engagement by both partners. Patriarchal norms are a barrier to condom negotiation by women in long-term relationships are they are not typically the “decision makers” of their households [25]. Women in concurrent, casual relationships have higher rates of condom uptake, suggesting that the expectation of monogamy, however unrealistic, along with the traditional structure of male partners holding authority pose challenges to male condom uptake [26]. This tension highlights the need for effective HIV prevention tools that do not rely on abstinence or “being faithful.” Our data support the concept that low rates of condom use are inexorably tied to public health messaging about condoms as a less desirable mode of HIV prevention.

**Fig. 1:**
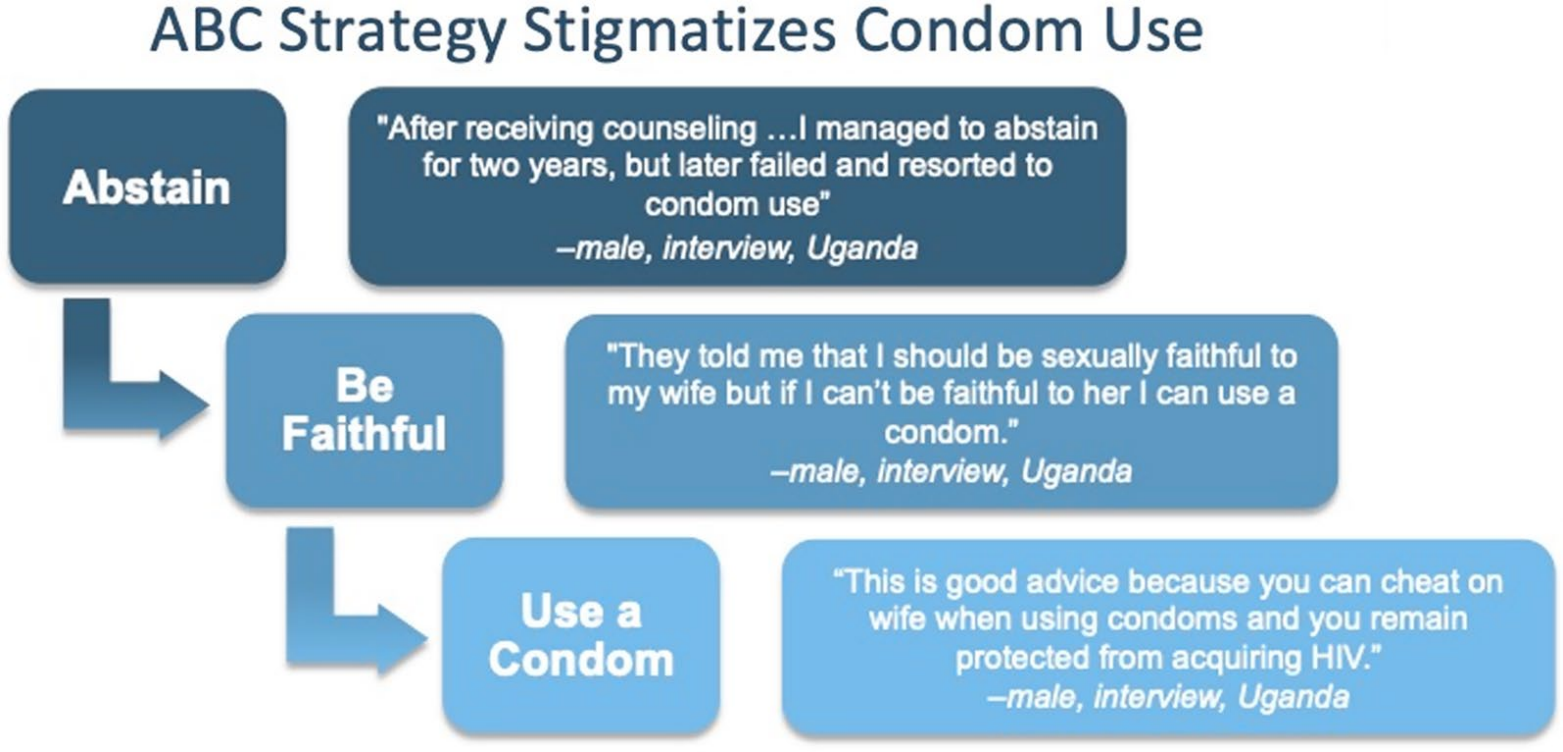
ABC public health messaging stigmatizes condom use through a hierarchical structure. Engagement with prevention at each level indicates failure at the level above

Stigmatization of condoms appears linked to the legacy of the ABC prevention model, whose messages persist. This model for HIV prevention is inherently hierarchical, where engagement at one level implies failure at the levels above it. Accordingly, abstinence is the best option for prevention, followed by being faithful, and finally by condom use. The ABC structure implies that, if someone is using condoms, they have failed to be abstinent and monogamous (Fig. 1). The hierarchy implied by the ABC model thereby creates a link between condom use and infidelity, thus stigmatizing the condom user.

### The legacy of ABC’s impact on new HIV prevention strategies

Understanding barriers to condom utilization can inform and improve prevention strategies in HIV-endemic communities. Our data show that the legacy of the ABC model is deeply entrenched. Male condom use within relationships is unlikely to increase so long as they are stigmatized as a marker of infidelity and partner distrust. Prevention campaigns that do not account for this stigma are likely to face significant barriers to uptake. Newer tools in HIV prevention, such as PrEP, may encounter similar challenges to uptake in long-term relationships as they too acknowledge risk of outside sexual partners, though they differ from condoms in that one partner could take PrEP without the other’s knowledge. The invisibility of PrEP could facilitate uptake by women who consider themselves to be at high risk but cannot safely have conversations about risk reduction in their relationships [27, 28]. Public health campaigns could address stigma earlier in the promotion of HIV prevention tools, which could allow for increased uptake without replicating the decades-long history of stigma that the male condom carries.

### Re-framing condom use

Though the prevention landscape has become more varied, male condom use remains an important tool in HIV prevention, family planning and STI prevention. We suggest that individual-centric messages may be culturally inappropriate for eastern Africa, where community, family, and religion may be valued above the individual [29]. An alternate message more aligned with prevailing social values may increase acceptability. Condoms’ unique position at the intersection of family planning and STI prevention could facilitate a change in messaging. For example, condoms could be ‘reframed’ to emphasize their utility as a method of family planning, or as a means of contributing positively towards the overall health of the community.

Reframing the conversation surrounding condom use to include family planning could empower women to negotiate condom use within relationships by de-centering mistrust [30]. Women in eastern Africa often believe that their husbands’ sexual behavior is their biggest source of HIV risk, and they lack control of their risk due to high incidence of extramarital sexual activity among married men [31, 32]. It is widely believed that women cannot realistically deny their husbands sex, and the stigma of male condom use within their partnerships leaves them vulnerable to HIV acquisition [10, 33]. While reframing condoms will not remove other barriers to uptake such as perceived decreased sexual pleasure, HIV prevention messages emphasizing condoms as family planning could allow women to more easily bring them into marriages, thus protecting both partners from HIV transmission.

### Removing hierarchy from HIV prevention

**Fig. 2:**
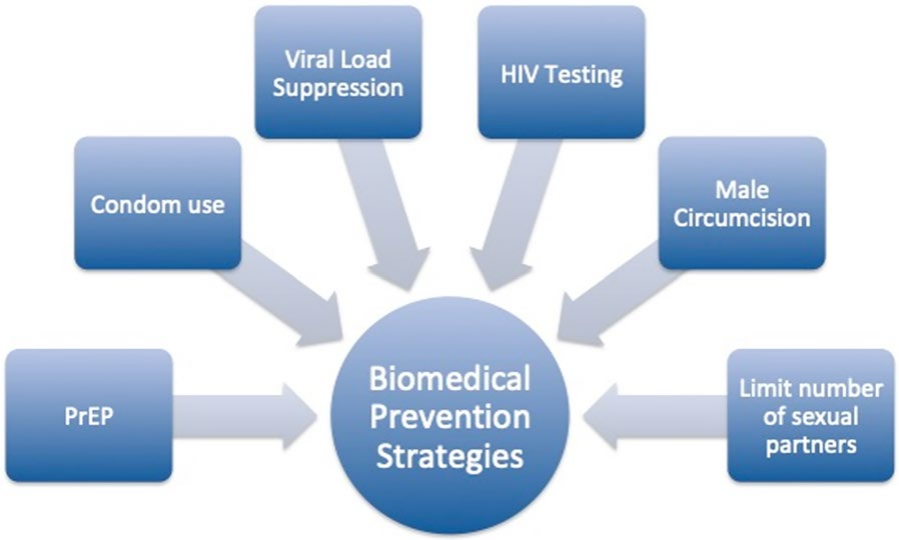
Combination prevention allows for non-hierarchical flexibility in prevention methods such that they can be customized for the community that they serve

We suggest that a non-hierarchical approach to HIV prevention may be an effective means to decrease HIV transmission. In an egalitarian model, multiple tools are valid and using one method does not signify failing at another: the goal is to pick one or more effective tools and use them (Fig. 2). “Combination prevention” strategies, like DREAMS’ packaged interventions, have successfully embraced this approach in health education campaigns throughout sub-Saharan Africa and achieved higher use of PrEP as HIV prevention, consistent male condom use, and lower rates of intimate partner violence [18, 34].

### Study strengths and limitations

This exploratory analysis was born from commonalities observed in the qualitative data collected in two separate studies. As one study used focus groups centered on family planning and the other utilized interviews on HIV prevention, our analysis is limited by the different aims, lines of questioning and data collection settings. While focus groups and interviews generate different forms of qualitative data, both have been shown as appropriate to elicit perspectives on stigmatized topics [35, 36]. Despite these differences in study design and conduct, the presence of consistent discussion of condom stigmatization in two different countries suggests that these observed perceptions may be widespread. A future, more formal exploration of this topic would likely illustrate a wider array of themes and could elucidate promising strategies to combat this stigma.

## Conclusions

The ABC prevention campaign is likely to have created negative perceptions of male condoms based on a hierarchical model that prioritizes abstinence and being faithful. In both Tanzania and Uganda, condoms are associated with infidelity and mistrust of sexual partners, which adversely impacts utilization. Public health interventions to increase condom uptake that do not address this stigma are likely to face significant barriers. To remove this stigma, HIV prevention messaging could be proactively reframed to a non-hierarchical approach in which condom use protects health of families and communities.

## Data Availability

The datasets used and/or analysed during the current study are available from the corresponding author on reasonable request.
